# Absence of Substantial Copy Number Differences in a Pair of Monozygotic Twins Discordant for Features of Autism Spectrum Disorder

**DOI:** 10.1155/2014/516529

**Published:** 2014-01-19

**Authors:** Marina Laplana, José Luis Royo, Anton Aluja, Ricard López, Damiàn Heine-Sunyer, Joan Fibla

**Affiliations:** ^1^Human Genetic Unit, Department of Basic Medical Sciences, University of Lleida, 25198 Lleida, Catalonia, Spain; ^2^Genetics of Complex Diseases Research Group, Biomedical Research Institute of Lleida (IRBLleida), 25198 Lleida, Catalonia, Spain; ^3^Biological-Factorial Models of Personality, Department of Psychology, University of Lleida, 25001 Lleida, Catalonia, Spain; ^4^Clinical Analysis Service, Universitari Arnau de Vilanova University Hospital, 25198 Lleida, Catalonia, Spain; ^5^Department of Genetics, Son Espases University Hospital, 07120 Palma de Mallorca, Spain

## Abstract

Autism spectrum disorder (ASD) is a highly heritable disease (~0.9) with a complex genetic etiology. It is initially characterized by altered cognitive ability which commonly includes impaired language and communication skills as well as fundamental deficits in social interaction. Despite the large amount of studies described so far, the high clinical diversity affecting the autism phenotype remains poorly explained. Recent studies suggest that rare genomic variations, in particular copy number variation (CNV), may account for a significant proportion of the genetic basis of ASD. The use of disease-discordant monozygotic twins represents a powerful strategy to identify *de novo* and inherited CNV in the disorder. Here we present the results of a comparative genome hybridization (CGH) analysis with a pair of monozygotic twins affected of ASD with significant differences in their clinical manifestations that specially affect speech language impairment and communication skills. Array CGH was performed in three different tissues: blood, saliva, and hair follicle, in an attempt to identify germinal and somatic CNV regions that may explain these differences. Our results argue against a role of large CNV rearrangements as a molecular etiology of the observed differences. This forwards future research to explore *de novo* point mutation and epigenomic alterations as potential explanations of the observed clinical differences.

## 1. Introduction

Autism spectrum disorder (ASD) is characterized by deficits in social interaction and social communication, as well as by the presence of repetitive behaviors, restricted interests, and particular speech impairments. Studies performed in siblings indicate that 85–90% of the ASD variability can be attributed to a genetic basis with a strong genotype-to-phenotype correlation. To date, whole genome association studies and exon sequencing in sporadic patients have revealed a plethora of candidate genes that explain a limited proportion of ASD heritability [1–4]. Copy number variants (CNVs) have been found to cause or predispose to ASDs [5, 6]. Previous works have identified multiple sporadic or recurrent CNVs, the majority of which occurred to be inherited from asymptomatic parents. Although highly penetrant CNVs or variants inherited in an autosomal recessive manner were detected in rare cases, previous results support the hypothesis that CNVs contribute to ASDs in association with other CNVs or point variants located elsewhere in the genome [5]. Several family history studies have demonstrated a strong familial background on language impairment [7]; however, their association as an endophenotype of ASD has not been systematically explored. Classical studies using a “broader autism phenotype” show a concordance rate of 92% for monozygotic twins and 10% for dizygotic twins [8]. In a more narrow ASD definition, concordance downs to 36% on monozygotic twins and 0% on dizygotic twins [9]. This later phenotypic discordance corresponds to twin pairs in which autism phenotype shows different degrees of the ASD manifestation. On a common genetic background predisposing to ASD, *de novo *germline and somatic mutations can differentially affect each twin and modify the ASD clinical manifestation. Newly developed strategies on genetic analysis such as the array comparative genomic hybridization (CGH) allow an in-depth exploration of the genomic structure of discordant siblings. In this work, we have taken advantage of array CGH to compare genomic DNA in three tissues: blood, saliva, and hair follicle, on a pair of discordant monozygotic twins with the aim to identify potential CNVs that could be associated with their differential ASD clinical outcomes.

## 2. Case Report

Subjects enrolled in the study were both 24-year-old male monozygotic twins denoted by TWO and TWX that were diagnosed with ASD at the age of 4. Parent consent and child assent were obtained prior to participating in this study. Their monozygosity was confirmed by concordance at SNP genotyping giving a probability of concordance by chance <10^−20^. Significant behavior differences were observed between both siblings since their childhood. TWO is entirely dependent on parental care and has serious mental retardation, serious deficiencies in language, and poor social interaction. In contrast, TWX completed basic education studies and professional training that allow workforce participation through social inclusion programs. While maintaining a parental support, this twin's capacity for interaction, including both language and social skills, is great. An in-depth characterization of the twins is presented in Tables 1–3. Autism spectrum diagnosis was assessed by the *Autism Diagnostic Interview-Revised* (ADI-R) [11] conducted with the parents of the referred twins and covers the subject's full developmental history (Table 1). Due to TWO's language limitations, the accompanying diagnostic test *Autism Diagnostic Observation Schedule* (ADOS) [12] could only be applied to TWX diagnoses. In spite of this, the Peabody Picture Vocabulary Test (PPVT-III) [13] was used to assess language capabilities on TWO. This test provides a quick estimate of verbal ability and scholastic aptitude of people who had mental retardation and reading or speech problems. Adaptive functioning was evaluated using the Vineland adaptive behavior scale (VABS) [14], a reliable test to measure a person's adaptive level of functioning at three domain structures: communication, daily living, and socialization (Table 2). The intelligence profile of the twins was assessed by two different tests: the *Leiter International Performance Scale-Revised* (Leiter-R) [15] applied to TWO and the *Wechsler Adult Intelligence Scale* (WAIS) [16] applied to TWX. Leiter-R test was devised to assess the intelligence of those with hearing or speech impairment being administered completely without the use of oral language, not even for instructions. All tests were performed by trained professional psychologist of the *Institut de Diagnòstic i Atenció Psiquiàtrica i Psicològica* (IDAPP) (Barcelona, Spain) and supervised by one of the coauthors (A. Aluja). A summary of the most relevant results obtained in the comparison of the twins' behaviour profile is presented in Table 3. Differences between twins on the communication and language dimension were evidenced. In addition, adaptive behavior course was clearly differentiable as reflected by twin's VABS scores.

According to the Diagnostic and Statistical Manual of Mental Disorders (4th edition) (DSM-IV) [17], TWO meets all criteria for a diagnosis of autistic disorder with moderate mental retardation. In contrast, the lack of stereotyped or repetitive behavior conducted to a diagnosis of pervasive developmental disorder not otherwise specified on TWX. Such clinical presentation contained significant differences that sustained a detailed genetic analysis. Our hypothesis was to consider that somatic mutations affecting CNVs would explain clinical differences. As somatic changes can arise randomly affecting different tissues, we tested three tissues with different embryological origins such as blood, coming from mesoderm, epidermal cells from saliva that has ectodermal origin, and hair follicle cells with ectodermal and neural crest origin [18]. Assuming that clinical difference arises from somatic CNV mutations that affect twin's neural development, the analyses of ectodermal/neural crest derived tissues are of interest.

Samples from blood, saliva, and hair follicle were used to perform a CGH analysis with the Agilent 400 K CGH array (Agilent Technologies, CA, USA) at Oxford Gene Technology facilities (Oxford, UK) according to the manufacturer's instructions. Genomic DNAs from each twin was compared to a reference obtained from a pool of DNAs from five healthy control males matched by age. In addition, a set of twin-to-twin comparisons was also performed. Copy number variation detection was conducted using CytoGenomics software (Agilent Technologies, CA, USA) adjusted at ADM2 algorithm threshold of 4.5 for the twin-to-twin comparisons and ADM2 algorithm threshold of 8 for the twin-to-reference comparisons. CNV regions (CNVR) were those including a minimum of 4 probes with significant *P* values. All genomic intervals are referred to as hg19. Graphical representation of data was done by Idiographica web server [10] and the Integrative Genome Viewer (IGV) [19]. DataBase of Genomic Variants (DBGV) was used as a source of the literature of described CNVs [21].

## 3. Results and Discussion

**Figure 1:**
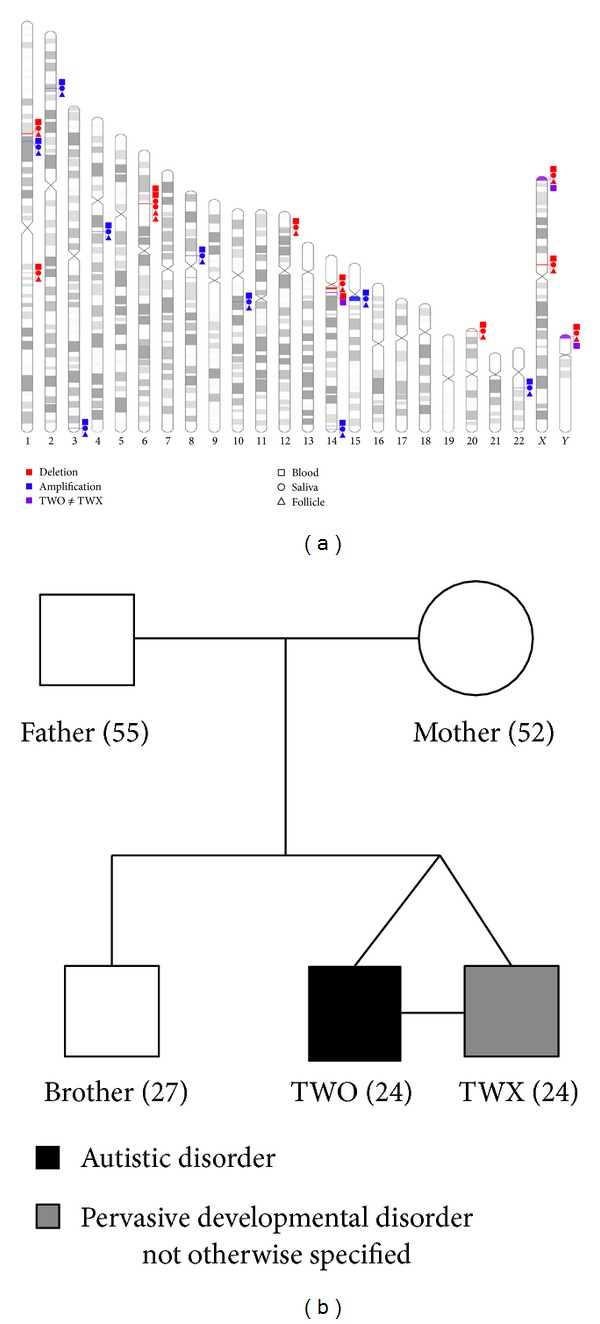
(a) Summary of copy number variant regions identified in this study plotted using *Idiographica* web server [10]. (b) Family pedigree of cases reported.

**Figure 2:**
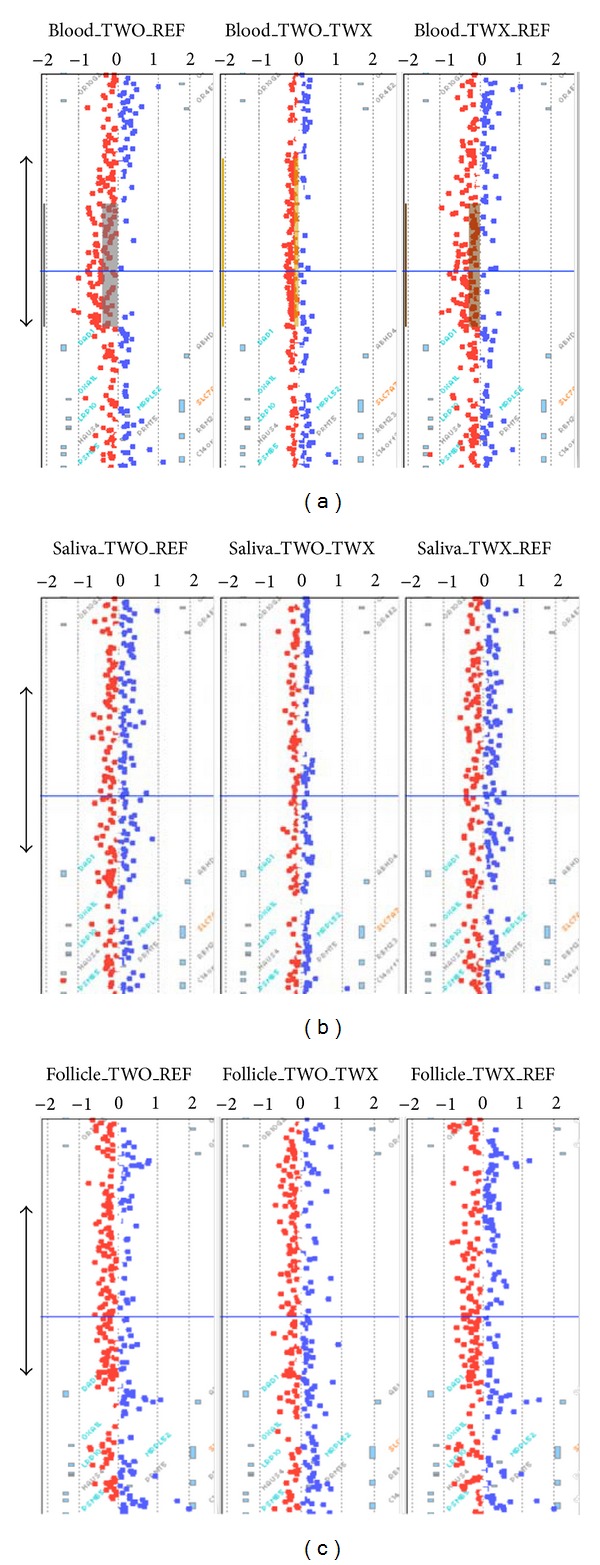
Screenshot of Agilent CytoGenomics software corresponding to chr14:22000000-23500000 region. (a) Blood, (b) saliva, and (c) hair follicle. On each panel (left to right) we present the CGH results from TWO-to-reference, twin-to-twin, and TWX-to-reference, respectively. Double arrows delimit the T-cell receptor alpha *locus*.

Pedigree examination did not reveal any family history of developmental delay (Figure 1). Normal karyotype analysis was observed in both twins and abnormalities at chromosome X affecting FMR1 *locus* methylation were discarded as an etiology for ASD. Results obtained from array CGH analyses are summarized in Figure 1 and Table 4. When twin's samples were compared to the reference pool a total of 19 CNVRs were identified. All but two were concordant regardless of the tissue analyzed. The first CNVR affecting chr14:22499836-22968425 shows a complex behavior. This CNVR was observed in both twin-to-twin and twin-to-reference comparisons of blood-derived DNA but neither in saliva nor in hair-follicle-derived DNA, evidencing the existence of somatic mosaicism within twins (Figure 2). A detailed analysis revealed that this region contains the T-cell receptor alpha *locus*, which has been associated with behavioral disturbances [22]. However, the fact that T-cell receptor alpha *locus* is subjected to V(D)J recombination and reported as a commonly *de novo* rearranged region among lymphoblastic cells leads us to be cautious about proposing the involvement of this region in the observed ASD differential outcomes.

The second CNVR with a differential behavior affected chrX:70397-2431564, which corresponds to the pseudoautosomal region of the X-Y chromosomes. This region contains an amplification detected in hair follicle DNA while deleted in blood and saliva tissues. Twin-to-twin comparison revealed amplification in TWO's blood but not in saliva nor in hair. On the other hand, twin-to-reference comparison showed a deletion in blood and saliva, while in hair follicle this region appears amplified. Given these unreliable results we considered the signal at this region not to be trustworthy and discarded it from further characterizations. Therefore, we could conclude that no significant differences in CNVRs between both twins could explain the observed differences on clinical profiles.

**Figure 3:**
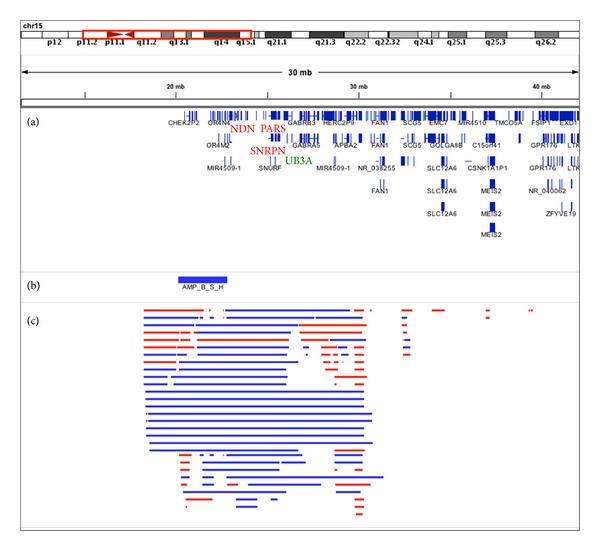
Screenshot of IGV [19] showing the 15q pericentromeric region. (a) Representation of the genes found in the vicinity. Those associated with Prader-Willi/Angelman Syndrome are highlighted in red/green, respectively. (b) Illustration of the duplicated region found in TWO and TWX. (c) Representation of the CNVRs from AUTDB previously associated with ASD (amplifications in blue and deletions in red) [20].

Although this is beyond the scope of the present study, we have explored CNV regions that may enlighten about twin's ASD etiology. All 19 regions detected overlap with previously described CNVRs at the DBGV [21]. From a total of 59 genes overlapping CNVRs, four (*HUWE1, TUBGCP5, ASMT,* and *PCDH15*) were found in AUTDB database [20] and to some extent were previously associated with ASD. Nevertheless, the most consistent CNVR with potential association with ASD was observed at chr15:20172544-22835945. This region of chromosome 15q11 was amplified in both twins when compared to reference (Figure 3). It should be noted that deletions but not amplifications on 15q11-q13 have been associated with Prader-Willi Syndrome. On the other hand, amplification affecting 15q11-q13 region has been associated with developmental disorders including autistic behavior [23]. However, our critical region is narrowed to 15q11 and does not include classical Prader-Willi Syndrome associated genes. In addition, CNVs that map within the 15q11.2 *locus* have been identified in autistic individuals in a number of reports. From genes mapping in this region, *NDN* (necdin, melanoma antigen (MAGE) family member), which is thought to be involved in the regulation of neuronal growth, and *CYFIP1* (cytoplasmic FMR1 interacting protein 1) are among the candidate genes proposed by the literature [24, 25]. *NDN* is distal to our critical region, while *CYFIP1* 5′ region is close to the CNVR breakpoint, running the possibility that *CYFIP1* regulatory landscape might be affected. Further analyses will be needed to explore this possibility.

Here we describe the CGH analysis on monozygotic twins affected with ASD but presenting significant clinical differences. Global analysis discarded the role of CNVs to explain these differences, since no variations have been found between both twins beyond the ones observed in the T-cell receptor region. CNV analysis of TWO>TWX in this region rather suggests lack of V(D)J recombination in TWO that should be explored in depth. Negative results should be viewed with caution until methodological limitations are ruled out as a possible explanation. In our case, the followed approach based on array CGH technology has demonstrated enough capability to detect genomic rearrangements affecting specific tissues such as the one we observe at the T-cell receptor alpha *locus*. This reflects that the approach followed in this study has enough sensibility to detect slight somatic differences between both twins. In addition, the detection of this tissue-specific mosaicism highlights the specificity and sensitivity of the array CGH methodology, ruling out methodological limitations as plausible explanation of our negative results. To date, different results can be found in the literature regarding the role of CNV in the etiological basis of disease discordance in monozygotic twins [26–28]. Results here presented are in line with previous works showing a lack of involvement of CNV in the discordant phenotype of monozygotic twins.

The characterization of the genetic etiology underlying the observed clinical differences between TWO and TWX must be addressed to explore *de novo* mutation events not detectable by CGH such as point mutations, as well as epigenomic alterations. Significant correlations between DNA methylation pattern and quantitatively measured autistic trait scores in concordant and discordant twin pairs have been found [29]. Further characterizations of TWO and TWX cases should be necessary to identify the causative genetic/epigenetic component to explain their discordant presentation of ASD.

## Figures and Tables

**Table 1:** Comparative evaluation of TWO and TWX according to Autism Diagnostic Interview-Revised (ADI-R).

	Threshold	TWO		TWX	
		Score	Observation	Score	Observation
Social reciprocal interaction	10	29	Significant	11	Significant
Communication skills	8	13	Significant	11	Significant
Behavior patterns restricted, repetitive, and stereotyped	3	7	Significant	1	Nonsignificant
Developmental difficulties observed before 36 months or less	1	5	Significant	3	Significant

**Table 2:** Comparative evaluation of TWO and TWX according to Vineland adaptive behavior scale (VABS).

Dimensions	TWO		TWX	
	Score	Equivalent age (years old)	Score	Equivalent age (years old)
Adaptive behavior composite	46		84	
Communication	20	1.6	65	3 to 11
Daily living	20	1.4	102	9 to 18
Socialization	20	0.8	96	9 to 18

**Table 3:** Summary results of the comparative behavioral evaluation of TWO and TWX.

Subject	Tests and measures	Evaluated dimensions				Diagnosis
		Communication and language	Socialization	Behavior patterns and interests	Intellectual ability^1^	
TWO	(i) Parents/subject interview (ii) PPVT-III (iii) Leiter-R (iv) ADI-R	(i) Strong limitations on communication and language (ii) Great degree of echolalia, repeating everything he says, even playing the same tone of voice (iii) Immediate echolalia of phrases that tell others, as well as echolalia of movie dialogues. Not maintaining reciprocal conversations with others (iv) Not manifesting through language and communicating his moods (v) Spontaneous verbal language not observed	(i) Enjoying verbal positive reinforcement (“good!,” “fantastic!,” “you are a champion!”) and physical (hitting hand or touching his back) (ii) Able to imitate descriptive gestures such as “hello,” “throw a kiss,” “ok,” and “water” and facial expressions and actions like cry, laugh, and “be happy” (iii) Able to recognize and pair “happy” and “sad” emotions in pictures and images	(i) Some stereotyped patterns of behavior (ii) Compulsive behavior by placing well and cleaning up his stuff (iii) Repetitive behavior to remove dirt from under his nails when talking or listening to others (iv) Compulsively cleaning drops that fall to the floor after showering	PPVT-III score: (i) IQ = 55 (ii) mental age equivalency = 3 years Leiter-R scores: (i) fluid reasoning scale: IQ = 48 (ii) spatial visual scale: IQ = 51	DSM-IV: autistic disorder with moderate mental retardation

TWX	(i) Parents/subject interview (ii) WAIS (iii) ADI-R (iv) ADOS	(i) No echolalia in language (ii) Good level of language that allows good communication skills (iii) Grammatical mistakes that sometimes require clarifications of their explanations (iv) Intonation is rather drab and accompanies his explanations by some stereotype words (v) Conversation with him is hardly mutual, providing information spontaneously but showing little interest in the interlocutor (vi) Nonverbal communication level is adequate, so their explanations are accompanied by gestures appropriately varied and integrated	(i) Social and responsible with their obligations (ii) Sympathetic to the people closest to him (iii) Poorly identifying his own difficulties and how these will interfere in his relations (iv) Eye contact is seen but rather intermittent (v) Facial expressions conveying emotions, some difficulty in identifying and describing by himself.	(i) No repetitive behavior observed	WAIS scores: (i) verbal IQ = 61 (ii) performance IQ = 82 (iii) full scale IQ = 69	DSM-IV: pervasive developmental disorder, not otherwise specified

^1^Range of normal scores = 80–120; average score population = 100.

PPVT-III: Peabody Picture Vocabulary Test. Leiter-R: Leiter International Performance Scale-Revised. ADI-R: Autism Diagnostic Interview-Revised. DSM-IV: Diagnostic and Statistical Manual of Mental Disorders, Fourth Edition. WAIS: Wechsler Adult Intelligence Scale. ADOS: Autism Diagnostic Observation Schedule.

**Table 4:** CNV regions (CNVR) distribution after array CGH comparisons of twin-to-twin (blood) and twin-to-reference (blood, saliva, and hair follicle).

Chr.	Start	End	Size	Number of probes	Twin-to-twin	Twin-to-reference						Number of CNVs at DBGV^1^	Genes contained in CNV region
						Blood		Saliva		Hair follicle	
						TWO	TWX	TWO	TWX	TWO	TWX
chr1	67955965	68093815	16	16		DEL	DEL	DEL	DEL	DEL	DEL	2	
chr1	72768855	72795480	26625	5		AMP	AMP	AMP	AMP	AMP	AMP	28	
chr1	152556449	152581944	25495	6		DEL	DEL	DEL	DEL	DEL	DEL	25	*LCE3C *
chr2	34697718	34738236	40518	8		AMP	AMP	AMP	AMP	AMP	AMP	12	
chr3	195421860	195444214	22354	9		AMP	AMP	AMP	AMP	AMP	AMP	28	*MIR570 *
chr4	69387056	69483277	96221	12		AMP	AMP	AMP	AMP	AMP	AMP	23	*UGT2B17, UGT2B15 *
chr6	32455274	32521929	66655	10		DEL	DEL	DEL	DEL	DEL	DEL	52	*HLA-DRB5, HLA-DRB6 *
chr6	32611013	32654142	43129	9		DEL	DEL	DEL	DEL	DEL	DEL	40	*HLA-DQA1, HLA-DQB1 *
chr8	39234992	39386158	151166	28		AMP	AMP	AMP	AMP	AMP	AMP	25	*ADAM5P, ADAM3A *
chr10	56448627	56468820	20193	5		AMP	AMP	AMP	AMP	AMP	AMP	9	***PCDH15***
chr12	9637323	9698517	61194	8		DEL	DEL	DEL	DEL	DEL	DEL	19	
chr14	19435611	20420849	985238	34		DEL	DEL	DEL	DEL	DEL	DEL	130	*POTEG, P704P, OR4Q3, OR4M1, OR4N2, OR4K2, OR4K5, OR4K1 *
chr14	22499836	22968425	468589	102	TWO > TWX	DEL	DEL	—	—	—	—	31	*TCR* alpha region
chr14	105401140	105431289	30149	8		AMP	AMP	AMP	AMP	AMP	AMP	4	*AHNAK2 *
chr15	20172544	22835945	2663401	110		AMP	AMP	AMP	AMP	AMP	AMP	282	*GOLGA6L6, GOLGA8C, BCL8, POTEB, NF1P1, LOC646214, CXADRP2, LOC727924, OR4M2, OR4N4, OR4N3P, GOLGA8D, GOLGA6L1, * ***TUBGCP5***
chr20	1563715	1580958	17243	5		DEL	DEL	DEL	DEL	DEL	DEL	27	*SIRPB1 *
chr22	24347959	24409603	61644	13		AMP	AMP	AMP	AMP	AMP	AMP	33	*LOC391322, GSTT1, GSTTP2, CABIN1 *
chrX (chrY)	70397	2431564	2361167	683	TWO > TWX^2^	DEL	DEL	DEL	DEL	AMP	AMP	106	*PLCXD1, GTPBP6, NCRNA00107, PPP2R3B, SHOX, CRLF2, CSF2RA, IL3RA, SLC25A6, NCRNA00105, ASMTL, P2RY8, SFRS17A, * ***ASMT*** *, DHRSX, ZBED1 *
chrX	53501375	53672366	170991	41		DEL	DEL	DEL	DEL	DEL	DEL	2	***HUWE1*** *, MIR98, MIRLET7F2 *

^1^Number of CNVs at Database of Genomic Variants (DBGV, [21]) overlapping CNVR detected.

^2^Amplified in TWO blood.

Highlighted in bold-italics genes previously associated with ASD according to AUTDB [20].

## References

[1] Shi L, Zhang X, Golhar R (2013). Whole-genome sequencing in an autism multiplex family. *Molecular Autism*.

[2] Liu L, Sabo A, Neale BM (2013). Analysis of rare, exonic variation amongst subjects with autism spectrum disorders and population controls. *PLoS Genetics*.

[3] Michaelson JJ, Shi Y, Gujral M (2012). Whole-genome sequencing in autism identifies hot spots for de novo germline mutation. *Cell*.

[4] Ghahramani Seno MM, Kwan BYM, Lee-Ng KKM (2011). Human PTCHD3 nulls: rare copy number and sequence variants suggest a non-essential gene. *BMC Medical Genetics*.

[5] Nava C, Keren B, Mignot C (2013). Prospective diagnostic analysis of copy number variants using SNP microarrays in individuals with autism spectrum disorders. *European Journal of Human Genetics*.

[6] Noh HJ, Ponting CP, Boulding HC (2013). Network topologies and convergent aetiologies arising from deletions and duplications observed in individuals with autism. *PLoS Genetics*.

[7] van Santen JPH, Sproat RW, Hill AP (2013). Quantifying repetitive speech in autism spectrum disorders and language impairment. *Autism Research*.

[8] Le Couteur A, Bailey A, Goode S (1996). A broader phenotype of autism: the clinical spectrum in twins. *Journal of Child Psychology and Psychiatry and Allied Disciplines*.

[9] Folstein S, Rutter M (1977). Genetic influences and infantile autism. *Nature*.

[10] Kin T, Ono Y (2007). Idiographica: a general-purpose web application to build idiograms on-demand for human, mouse and rat. *Bioinformatics*.

[11] Lord C, Rutter M, Couteur AL (1994). Autism diagnostic interview-revised: a revised version of a diagnostic interview for caregivers of individuals with possible pervasive developmental disorders. *Journal of Autism and Developmental Disorders*.

[12] Lord C, Rutter M, Goode S (1989). Autism diagnostic observation schedule: a standardized observation of communicative and social behavior. *Journal of Autism and Developmental Disorders*.

[13] Dunn LM, Douglas MD (2007). *Peabody Picture Vocabulary Test*.

[14] Sparrow SS, Cicchetti DV, Balla DA (2005). *Vineland Adaptive Behavior Scales*.

[15] Roid GH, Miller LJ, Roid GH, Miller LJ (1997). Leiter international performance scale—revised: examiner’s manual. *Leiter International Performance Scale-Revised*.

[16] Wechler D (2008). *Wechsler Adult Intelligence Scale (WAIS)*.

[17] American Psychiatric Association (2000). *Diagnostic and Statistical Manual of Mental Disorders*.

[18] Scott FG, Susan RS, Mary ST, Ronald NK (2000). *Developmental Biology*.

[19] Robinson JT, Thorvaldsdóttir H, Winckler W (2011). Integrative genomics viewer. *Nature Biotechnology*.

[20] Basu SN, Kollu R, Banerjee-Basu S (2009). AutDB: a gene reference resource for autism research. *Nucleic Acids Research*.

[21] Iafrate AJ, Feuk L, Rivera MN (2004). Detection of large-scale variation in the human genome. *Nature Genetics*.

[22] Hallmayer J, Faraco J, Lin L (2009). Narcolepsy is strongly associated with the T-cell receptor alpha locus. *Nature Genetics*.

[23] Swanwick CC, Larsen EC, Banerjee-Basu S, Deutsch S (2011). Genetic heterogeneity of autism spectrum disorders. *Autism Spectrum Disorders: The Role of Genetics in Diagnosis and Treatment*.

[24] Chibuk TK, Bischof JM, Wevrick R (2001). A necdin/MAGE-like gene in the chromosome 15 autism susceptibility region: expression, imprinting, and mapping of the human and mouse orthologues. *BMC Genetics*.

[25] de Rubeis S, Bagni C (2011). Regulation of molecular pathways in the fragile X syndrome: insights into autism spectrum disorders. *Journal of Neurodevelopmental Disorders*.

[26] Bloom RJ, Kähler AK, Collins AL (2013). Comprehensive analysis of copy number variation in monozygotic twins discordant for bipolar disorder or schizophrenia. *Schizophrenia Research*.

[27] Ehli EA, Abdellaoui A, Hu Y (2012). De novo and inherited CNVs in MZ twin pairs selected for discordance and concordance on attention problems. *European Journal of Human Genetics*.

[28] Ono S, Imamura A, Tasaki S (2010). Failure to confirm CNVs as of aetiological significance in twin pairs discordant for schizophrenia. *Twin Research and Human Genetics*.

[29] Wong CY, Meaburn EL, Ronald A (2013). Methylomic analysis of monozygotic twins discordant for autism spectrum disorder and related behavioural traits. *Molecular Psychiatry*.

